# Artificial intelligence tool utilization in deprescribing practices among community pharmacists in Jordan: a cross-sectional study

**DOI:** 10.3389/fdgth.2026.1876269

**Published:** 2026-08-24

**Authors:** Ahmad Z. Al Meslamani, Dania Abu-Naser, Anan S. Jarab, Khadeijah Ahmed Al Marshoodi, Nada A. Alsaleh

**Affiliations:** 1 College of Pharmacy, Al Ain University, Abu Dhabi, United Arab Emirates; 2 Department of Applied Health Sciences, Irbid University College, Al-Balqa Applied University, Irbid, Jordan; 3 Department of Clinical Pharmacy, Faculty of Pharmacy, Jordan University of Science and Technology, Irbid, Jordan; 4 College of Pharmacy, University of Sharjah, Sharjah, United Arab Emirates; 5 Department of Pharmacy Practice, College of Pharmacy, Princess Nourah Bint Abdulrahman University, Riyadh, Saudi Arabia

**Keywords:** AI, deprescribing, Jordan, medication review, polypharmacy

## Abstract

**Introduction:**

Data on artificial intelligence-supported deprescribing in Jordanian community pharmacy are still lacking. This study aimed to assess current deprescribing practices among community pharmacists in Jordan, identify the medication classes most commonly targeted for deprescribing, evaluate perceived outcomes, and determine factors associated with AI tool use in deprescribing.

**Methods:**

This was a cross-sectional survey-based study conducted among licensed community pharmacists in Jordan from 15 November 2025 to 15 March 2026 using online, in-person, and telephone recruitment. The questionnaire assessed participant and practice characteristics, deprescribing activities, digital infrastructure, and use of AI tools. For the primary analysis, AI use was operationalized specifically as self-reported AI-chatbot use, whereas other computerized decision-support tools were reported separately. The SPSS (version 29) was used to conduct descriptive statistics, chi-square tests, and multivariable logistic regression.

**Results:**

Of the 1,036 pharmacists who completed the questionnaire, 798 (77.0%) reported practicing deprescribing. The medication classes most frequently reported as targets of deprescribing-related activity were antibiotics (799, 77.1%), proton-pump inhibitors (790, 76.3%), vitamins and mineral supplements (776, 74.9%), non-opioid analgesics/nonsteroidal anti-inflammatory drugs (753, 72.7%), and antidiabetic drugs (638, 61.6%). More than half of the pharmacists (641, 61.9%) reported using a digital tool to identify deprescribing opportunities and (340, 32.8%) reported using AI chatbot. Frequently observed outcomes after deprescribing were better patient-reported health (429, 41.4%), improved adherence (417, 40.3%), and fewer side effects (417, 40.3%), whereas worsening of symptoms was reported less often (89, 8.6%). AI use was independently associated with male gender (adjusted odds ratio [AOR] 1.32, 95% confidence interval [CI] 1.01–1.73), PharmD qualification versus BPharm/BSc (AOR 1.38, 95% CI 1.02–1.87), urban practice versus rural practice (AOR 1.52, 95% CI 1.04–2.22), and reliable internet access (AOR 3.89, 95% CI 1.95–7.76).

**Conclusion:**

Deprescribing is widely reported in Jordanian community pharmacy practice, and one-third of pharmacists use AI chatbots to support deprescribing decisions. Strengthening internet access, targeted training, and equitable infrastructure may help expand AI-assisted deprescribing across practice settings.

## Introduction

Polypharmacy and potentially inappropriate medication use have become major challenges in chronic disease management (1). It is associated with adverse drug events, cognitive and functional impairment, falls, hospitalization, mortality, and increased health-care costs (2, 3). In this regard, deprescribing, which is the supervised, patient-centred process of identifying and reducing or discontinuing medicines when their actual or potential harms outweigh their likely benefits, has emerged not as the opposite of prescribing, but as an essential component of high-quality prescribing (4, 5).

Deprescribing requires medication review, individualized benefit-risk assessment, shared decision-making, and follow-up (6). A recent study systematic review found that deprescribing interventions in community-dwelling older adults can reduce overall medication burden (5). Another review reported that pharmacist-led and community-based deprescribing interventions have been associated with greater medication discontinuation and financial benefit, even if effects on mortality, falls, hospitalization, and quality of life remain less consistent across studies (7).

Pharmacy practice is undergoing a rapid transition toward digitally supported models of care. Artificial intelligence (AI) applications in pharmacy include prescription screening, medication-related problems identification, information retrieval and synthesis, adverse-event risk estimation, adherence support, workflow optimization, and flagging potential medication-review or deprescribing opportunities (4, 8). Only seven qualifying studies of current AI application in pharmacy practice were found in a recent scoping review (8).

Evidence specifically on pharmacists’ use of AI for medication review and deprescribing remains at an early stage. A recent study developed GPT-4-based chatbot that facilitated the deprescribing of proton pump inhibitors in cystic fibrosis patients by providing tailored, accessible education that addresses communication barriers without increasing clinician workload (9). Additionally, a scoping review of AI applications in geriatric care indicated that these tools significantly enhance medication safety by accurately identifying drug-drug interactions, detecting potentially inappropriate medications, and improving treatment adherence through user-friendly interfaces (10). Despite these primary promising news, a retrospective cohort study found that while large language models (LLMs) significantly outperformed medical students in filtering relevant deprescribing criteria in emergency settings, they struggled to provide accurate final recommendations due to the complexity of specific clinical contexts and poorly calibrated confidence levels (11).

In Jordan, Jordanian pharmacy AI studies have examined general attitudes toward AI, informatics, or ChatGPT rather than deprescribing-specific use. Community pharmacists have shown support for the implementation of AI, but they have identified many significant obstacles, including as the scarcity of AI-related gear and software, the need for human supervision, and running costs (12). Another study reported that pharmacists in Jordan generally acknowledged ChatGPT potential educational and informative usefulness, but over half had never used it in practice, and worries about bias, inaccuracy, privacy, and legal ramifications were prevalent (13). The Jordanian literature on deprescribing is itself still limited. A narrative review identified only 10 Jordanian studies addressing deprescribing or polypharmacy, and local work has focused mainly on prevalence studies, potentially inappropriate prescribing, and attitudes among patients or students rather than on the real-world deprescribing activities of community pharmacists (14). The literature search revealed data on AI-supported deprescribing in Jordanian community pharmacy are still lacking. Addressing this gap is important because community pharmacists are accessible medication professionals who may become key end-users of emerging decision-support technologies in everyday medication review and deprescribing practice.

Therefore, this study aimed to describe the frequency and characteristics of deprescribing-related activities in community practice, identify commonly targeted medication classes and self-reported outcomes following deprescribing, determine pharmacists’ awareness, current use, and comfort with AI or digital decision-support tools, while explicitly distinguishing self-reported AI-chatbot use from other computerized decision-support tools; and identify demographic, professional, and practice-setting factors independently associated with AI-chatbot use in deprescribing-related practice.

## Methodology

### Study design and setting

This was a cross-sectional quantitative survey of community pharmacists in Jordan. Data were collected using non-probability convenient sampling over a four-month period (15 November 2025 to 15 March 2026) through web-based platforms, in-person interviews, and telephone calls. The design and reporting of the online questionnaire were informed by the Checklist for Reporting Results of Internet E-Surveys (CHERRIES) (15). The STROBE checklist for cross-sectional studies was also adopted to improve the methodological approaches of the study (16).

### Eligibility criteria for participants

Officially licensed pharmacists who were actively practicing in community setting in Jordan comprised the research population. Eligibility was carefully restricted to those with at least six months of continuous practice experience to provide sufficient professional exposure. Pharmacy managers, owners, and full-time and part-time staff pharmacists made up the cohort. Pharmacists working only in non-community settings, trainees and supervised interns were not included. Recruitment included pharmacists from Amman and the northern, central, and southern governorates to broaden geographic coverage. Because participants were not selected from a probability-based sampling frame, the sample was not designed to be nationally representative.

### Ethics approval

The research protocol (IRB No. 2026/2025/2/45) was reviewed and approved by the Ethics Committee of Al-Balqa Applied University in Jordan. Participation was strictly voluntary. The initial page of the online survey contained a participant information statement that delineated the purpose of the research, the anonymous nature of participation, the prospective use of the data, and the ability to withdraw from the survey at any point before submission. Prior to respondents proceeding to the questionnaire, electronic informed consent was obtained. No personally identifiable information was collected.

### Sample size

Using the most recent accessible JPA-based estimate of 7,525 practicing community pharmacists in Jordan (17), and using the following formula (18): N is the total population size, r is the anticipated proportion (as a percentage) for the outcome of interest, Z is a critical value for chosen confidence interval (95%), Z≈1.96. E is the margin of error (5%). *n* is the calculated sample size.

$$Z\left(\frac{c}{100}\right)2\times r\;(100-r),\;n=\;\frac{N\;\times x}{(N-1)\times E^{2}+x\;},\;E=\;\sqrt{\frac{(N-n)x}{n\;(N-1)}}$$

The minimum recruitment targetwas 366 pharmacists. A total of 1,036 complete questionnaires were included to support estimate stability and the planned subgroup analyses. Because recruitment used non-probability convenience sampling, exceeding the minimum target did not eliminate selection bias or confer national representativeness.

### Study instruments

In this study, the questionnaire was derived from various published sources following an exhaustive literature analysis since no single questionnaire adequately addressed community pharmacists’ deprescribing practice and AI-enabled decision assistance. The validated Comprehensive Healthcare Providers’ Opinions, Preferences, and Attitudes towards Deprescribing (CHOPPED) questionnaire informed the deprescribing component, while previous healthcare-professional surveys supplied items on deprescribing tool awareness and factors influencing deprescribing. The digital-health and artificial-intelligence questions were modified from Jordanian community pharmacy surveys on AI attitudes, readiness, previous experience, and hurdles to informatics and technology adoption in pharmacy (12, 19–21).

The final instrument included four sections and 25 core items. The first section assessed participants’ gender, pharmacy region, highest pharmacy qualification, years of community pharmacy experience, and current position. The second section examined the practice environment, workload, and digital infrastructure, including pharmacy type and location, daily patient and prescription volume, private counselling area, computer/tablet and internet access, electronic prescription or patient-record systems, and use of online clinical resources during patient care. The third section examined current deprescribing practices, including medication reviews, the estimated proportion of patients with at least one medication deprescribed in the previous year, physician consultation, percieved outcomes, and the medication classes most often targeted for deprescribing. Medication class items included proton-pump inhibitors, benzodiazepines/Z-drugs, antidepressants, antipsychotics, lipid-lowering agents, antihypertensives, antidiabetic drugs, opioid and non-opioid analgesics/NSAIDs, antiplatelets/anticoagulants, thyroid medications, inhalers, systemic corticosteroids, antibiotics, osteoporosis medications, hormonal contraceptives/hormone replacement therapy, and vitamins/mineral supplements. The fourth section assessed evaluated familiarity with software-assisted drug review, knowledge of the use of digital tools for deprescribing, and the particular tools used. The major AI category was limited to self-reported usage of an AI chatbot for reporting and analytical purposes. The usage of AI chatbots was not included in the main outcome; instead, MedSafer, Beers Criteria apps, STOPP/START-based tools, and other structured electronic resources were reported separately as additional computerized decision-support aids. AI chatbots and other computerized help were included under the general phrase “digital tools.” The underlying technological architecture of the claimed chatbots was not independently verified by the questionnaire. Pharmacists were asked to choose outcomes they had seen or felt after deprescribing using a study-specific multiple-response checklist. It was not a validated tool for patient-reported outcomes. Responses were not independently checked, evaluated at a predetermined follow-up period, or connected to specific patients or therapies. While the majority of the items utilized closed-ended category or ordinal response styles, certain questions on perceived results, digital tools, and pharmaceutical classifications permitted multiple answers.The questionnaire was developed and administered in English rather than Arabic because English is the official language for studying medical sciences, including pharmacy, in Jordan (22). Before dissemination, the draft questionnaire underwent face and content review by 5 experts in clinical pharmacy, community pharmacy practice, geriatric pharmacotherapy, and health informatics, who assessed item relevance, clarity, comprehensiveness, and contextual appropriateness. Based on their feedback, minor wording and sequencing changes were made. These changes include the deprescribing-tool and AI-related items. The revised instrument was then piloted among 15 community pharmacists to assess readability, item flow, and average completion time. Responses from the pilot testing were excluded from the final analysis.

### Data collection

A total of 25 trained data collectors, including trainee pharmacy students and research team members, assisted with participant recruiting and survey administration. The questionnaire was created using Google Forms. In order to collect data in-person, training students visited community pharmacies, explained the project, verified eligibility, and asked pharmacists to take part. Additionally, eligible pharmacists were contacted by phone to explain the research and make it easier for them to complete the survey. To increase distribution, the survey link was also sent electronically via the social media networks. Specifically, we used the Facebook, Telegram, and WhatsApp groups of Jordanian pharmacists. To increase participation, reminders were sent out on a regular basis throughout the data collecting period.

A specific denominator for all pharmacists who got the invitation could not be determined since recruiting depended on many overlapping channels, such as in-person interactions, phone outreach, online professional groups, and peer sharing. Prior to analysis, every completed record was checked for internal consistency, eligibility, and fulfilment of the essential elements utilised in this work. For respondents who said they did not utilise digital tools, the conditional follow-up question on the particular digital tools used for deprescribing was considered fundamentally lacking. Rather than being interpreted on the respondent's behalf, the impacted item was classified as missing when blatantly contradicting checkbox-grid replies were found during data cleaning.

### Data analysis

Data were exported from Google Forms into Microsoft Excel and then analysed using IBM SPSS Statistics for Windows, version 29.0 (IBM Corp., Armonk, NY, USA). Checking variable ranges, harmonising duplicate answer labels, spotting improbable or ambiguous inputs in the free-text numeric fields, and looking for internally inconsistent checkbox-grid replies were all part of the data cleaning process. Frequencies and percentages were used to summarise categorical variables. Before descriptive analysis, multiple-response items, that is, observable outcomes after deprescribing and particular digital tools used to discover deprescribing opportunities, were broken down into binary indicators; as a result, percentages for these items were permitted to surpass 100%.

Additionally, respondents were stratified according to whether they reported practicing deprescribing in their current community pharmacy role and whether they reported using AI in deprescribing-related medication review. In order to differentiate AI-enabled support from non-AI digital resources, tools were categorized based on their fundamental purpose. MedSafer was seen as a specialized rule-based electronic clinical decision support system that creates customized deprescribing reports by applying encoded evidence-based deprescribing criteria to patient and pharmaceutical data. Instead of being seen as AI chatbots, Beers Criteria apps and STOPP/START-based tools were viewed as electronic implementations of explicit expert-derived screening criteria; that is, traditional electronic references or rule-based decision-support aids, depending on the implementation. Self-reported usage of any AI chatbot among the tools used to find possible deprescribing possibilities was conservatively operationalized as AI use in deprescribing for the main study. AI users did not include respondents who solely chose MedSafer, a Beers Criteria application, a STOPP/START-based tool, or any other non-AI digital resource. The two binary practice outcomes and categorical participant factors were compared using Pearson's chi-square test.

The usage of AI in deprescribing was then fitted as the dependent variable in a multivariable binary logistic regression model. Adjusted odds ratios (AORs) with 95% confidence intervals (CIs) were estimated by entering factors with *P* < 0.30 in the bivariate analyses into the multivariable model in accordance with the analytical methodology defined for this paper. Interpretability and category size were taken into consideration while choosing reference categories. A *P* value of less than 0.05 was regarded as statistically significant in all two-sided statistical tests.

## Results

### Participant and practice-setting characteristics

A total of 1,036 community pharmacists were included in this study, of which 719 (69.4%) were female, 555 (53.6%) held a BPharm/BSc Pharmacy degree, and 714 (68.9%)practiced in the northern governorates (714, 68.9%) (Table 1). Independent private pharmacies constituted the predominant practice setting, reported by 633 (61.1%), while 422 (40.7%) and 415 (40.1%) participants worked in semi-urban and urban locations, respectively (Table 2**).** Of the included pharmacists, 741 (71.5%) reported the availability of a private counselling area in their pharmacies, 723 (69.8%) reported reliable internet access during working hours, and 746 (72.0%) reported using an electronic system for at least some prescriptions or patient records.

**Table 1:** Participants’ demographics, qualifications, and career background (*N* = 1,036).

Characteristic	Total *n* (%)	Using an AI chatbot in deprescribing Yes (*n* = 340) *n* (%)	Using an AI chatbot in deprescribing No (*n* = 696) *n* (%)	*P*-value
Gender				
Female	719 (69.4)	223 (65.6)	496 (71.3)	0.09
Male	317 (30.6)	117 (34.4)	200 (28.7)	
Region of pharmacy location				
Amman	197 (19.0)	77 (22.6)	120 (17.2)	0.24
Central governorates	90 (8.7)	31 (9.1)	59 (8.5)	
Northern governorates	714 (68.9)	222 (65.3)	492 (70.7)	
Southern governorates	35 (3.4)	10 (2.9)	25 (3.6)	
Highest pharmacy-related qualification				
BPharm/BSc Pharmacy	555 (53.6)	163 (47.9)	392 (56.3)	0.08
MSc in Pharmacy/Clinical Pharmacy	116 (11.2)	45 (13.2)	71 (10.2)	
Other health-related degree	92 (8.9)	27 (7.9)	65 (9.3)	
PharmD	220 (21.2)	83 (24.4)	137 (19.7)	
PhD in Pharmacy/Pharmaceutical Sciences	53 (5.1)	22 (6.5)	31 (4.5)	
Years working in community pharmacy				
No experience/not currently working	66 (6.4)	17 (5.0)	49 (7.0)	0.78
< 1 year	40 (3.9)	13 (3.8)	27 (3.9)	
1 to <3 years	361 (34.8)	126 (37.1)	235 (33.8)	
3 to <6 years	305 (29.4)	104 (30.6)	201 (28.9)	
6–10 years	137 (13.2)	43 (12.6)	94 (13.5)	
> 10 years	93 (9.0)	27 (7.9)	66 (9.5)	
Other/unclear	34 (3.3)	10 (2.9)	24 (3.4)	
Current position in the pharmacy				
Full-time staff pharmacist	355 (34.3)	107 (31.5)	248 (35.6)	0.88
Intern/trainee pharmacist	97 (9.4)	34 (10.0)	63 (9.1)	
Part-time staff pharmacist	236 (22.8)	75 (22.1)	161 (23.1)	
Pharmacy manager (not owner)	177 (17.1)	64 (18.8)	113 (16.2)	
Pharmacy owner	171 (16.5)	60 (17.6)	111 (15.9)	

Data are presented as *n* (%). *P*-values calculated using chi-square test. Bold *P*-values indicate statistical significance (*P* < 0.05).

**Table 2:** Participants’ practice environment, workload, and electronic system usage (*N* = 1,036).

Characteristic	Total *n* (%)	Using an AI chatbot in deprescribing Yes (*n* = 340) *n* (%)	Using an AI chatbot in deprescribing No (*n* = 696) *n* (%)	*P*-value
Type of community pharmacy				
Chain pharmacy (national or regional chain)	244 (23.6)	88 (25.9)	156 (22.4)	0.44
Governmental/public community pharmacy	109 (10.5)	33 (9.7)	76 (10.9)	
Independent private pharmacy	633 (61.1)	201 (59.1)	432 (62.1)	
Pharmacy inside a private polyclinic or outpatient medical center	50 (4.8)	18 (5.3)	32 (4.6)	
Area where the pharmacy is located				
Rural	199 (19.2)	53 (15.6)	146 (21.0)	**0** **.** **03**
Semi-urban	422 (40.7)	134 (39.4)	288 (41.4)	
Urban	415 (40.1)	153 (45.0)	262 (37.6)	
Patients/customers served per working day				
< 50/day	519 (50.1)	152 (44.7)	367 (52.7)	0.21
50–99/day	348 (33.6)	118 (36.8)	223 (32.0)	
100–199/day	120 (11.6)	44 (12.9)	76 (10.9)	
>=200/day	49 (4.7)	19 (5.6)	30 (4.3)	
Prescription medicines dispensed per working day				
< 25/day	540 (52.1)	163 (47.9)	377 (54.2)	**0**.**09**
25–49/day	272 (26.3)	92 (27.1)	180 (25.9)	
50–99/day	139 (13.4)	51 (15.0)	88 (12.6)	
100–199/day	53 (5.1)	21 (6.2)	32 (4.6)	
>=200/day	32 (3.1)	13 (3.8)	19 (2.7)	
Private counselling area available				
Yes	741 (71.5)	261 (76.8)	480 (69.0)	**0**.**01**
No	295 (28.5)	79 (23.2)	216 (31.0)	
Computer/tablet with internet access during working hours				
Reliable internet available at all times	723 (69.8)	268 (78.2)	455 (65.4)	**<0**.**001**
Internet available but often unstable or slow	159 (15.3)	47 (13.8)	112 (16.1)	
Computer/tablet available without internet	76 (7.3)	15 (4.4)	61 (8.8)	
No computer/tablet available	78 (7.5)	10 (2.9)	68 (9.8)	
Electronic system for prescription processing or patient records				
Yes, for all prescriptions	388 (37.5)	157 (46.2)	231 (33.2)	0.13
Yes, for some prescriptions	358 (34.6)	117 (34.4)	241 (34.6)	
No, all prescriptions processed on paper	290 (28.0)	66 (19.4)	224 (32.2)	
Use of online clinical resources during patient care				
Several times per day	324 (31.3)	138 (40.6)	186 (26.7)	0.21
A few times per week	389 (37.5)	136 (40.0)	253 (36.4)	
Rarely	246 (23.7)	56 (16.5)	190 (27.3)	
Never	77 (7.4)	10 (2.9)	67 (9.6)	

Data are presented as *n* (%). *P*-values calculated using chi-square test. Bold *P*-values indicate statistical significance (*P* < 0.05).

### Deprescribing practices

**Figure 1:**
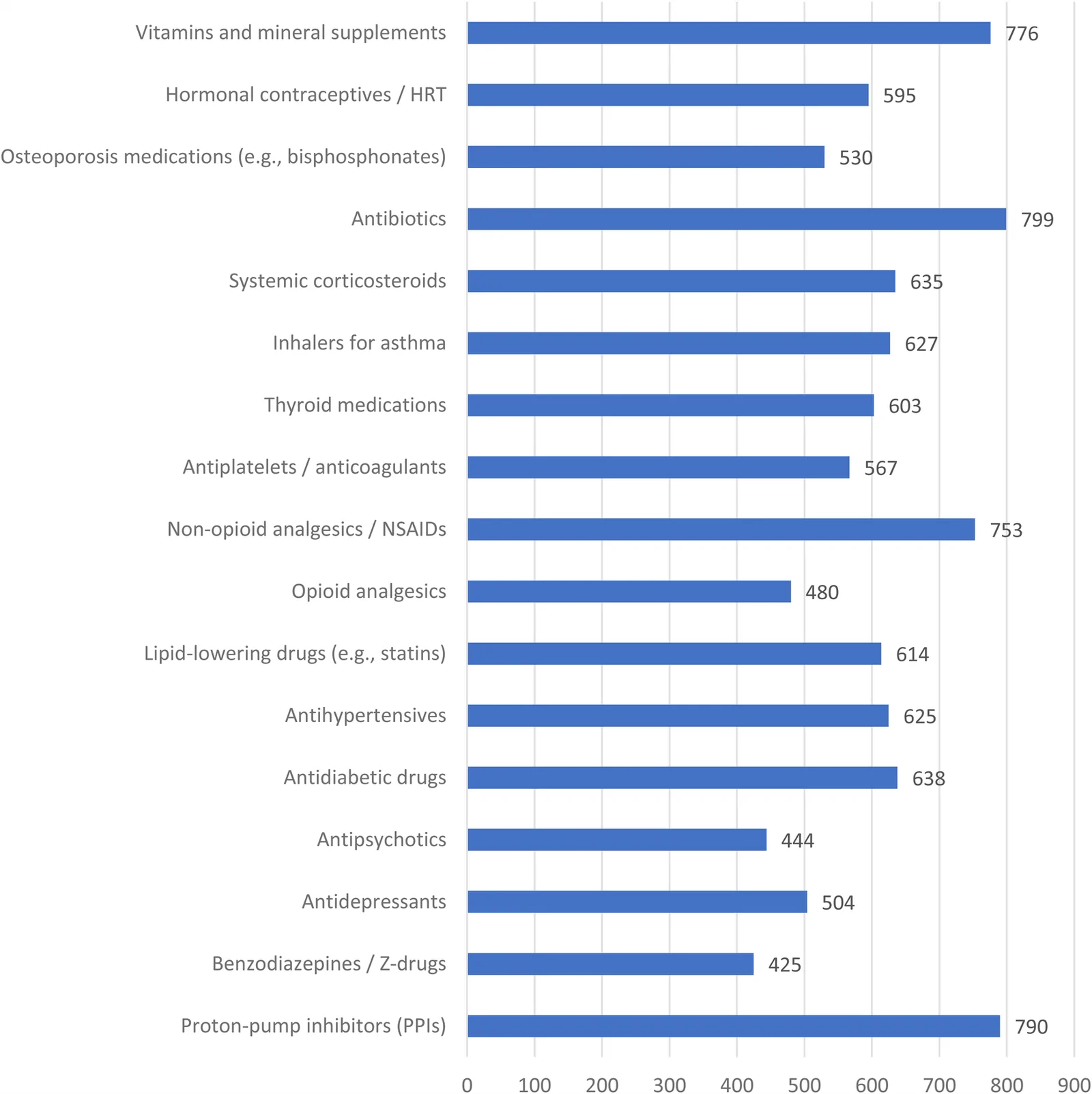
Medication classes most frequently reported as targets of deprescribing-related activity.

Overall, 798 (77.0%) pharmacists reported that they practiced deprescribing. In the bivariate analysis, Participant demographics, qualifications, and work experience did not differ significantly between pharmacists who reported using an AI chatbot in deprescribing and those who did not (all *p* > 0.05; Table 1). The medication classes most frequently reported as targets of deprescribing-related activity were antibiotics, reported by 799 (77.1%), proton-pump inhibitors, reported by 790 (76.3%), vitamins and mineral supplements, reported by 776 (74.9%), non-opioid analgesics/NSAIDs, reported by 753 (72.7%), and antidiabetic drugs, reported by 638 (61.6%) **(**Figure 1**).**

Across participating pharmacists, 463 (44.7%) had completed continuing professional development related to geriatrics, polypharmacy, or deprescribing during the preceding 12 months, whereas 565 (54.5%) had completed training related to digital health, clinical decision support, or artificial intelligence tools (Table 3). Overall, 641 (61.9%) reported currently using at least one a digital tool to identify deprescribing opportunities; 340 (32.8%) reported using an AI chatbot for deprescribing and 217 (20.9%) reported using MedSafer. Because respondents could select more than one tool, these categories were not mutually exclusive. Interprofessional collaboration was common, with 402 (38.8%) reporting that they always consulted the patient's physician during deprescribing and a further 339 (32.7%) reporting that they often did so. Outcomes were measured using a multiple-response item asking pharmacists what they had observed or perceived after deprescribing. The most frequently reported outcomes following deprescribing were better patient-reported health, reported by 429 (41.4%), improved adherence, reported by 417 (40.3%), and fewer side effects, reported by 417 (40.3%). Worsening of symptoms was reported by 89 (8.6%). These figures represent pharmacists’ self-reports and were not independently verified or causally attributed to deprescribing.

**Table 3:** Deprescribing practice, digital tool use, observed outcomes, and medication classes frequently deprescribed (*N* = 1,036).

Characteristic/response	Total, *n* (%)
CPD related to geriatrics, polypharmacy, or deprescribing in the last 12 months	
Yes	463 (44.7)
No	573 (55.3)
CPD related to digital health, clinical decision support, or AI tools in the last 12 months	
Yes	565 (54.5)
No	471 (45.5)
Frequency of medication reviews aimed at deprescribing	
Daily	363 (35.0)
Weekly	329 (31.8)
Monthly	200 (19.3)
Seldom	115 (11.1)
Never	29 (2.8)
Proportion of patients with >=1 medication deprescribed in the past year	
<10%	431 (41.6)
10–25%	404 (39.0)
26–50%	144 (13.9)
>50%	57 (5.5)
Observed outcomes after deprescribing	
Improved adherence	417 (40.3)
Fewer side effects	417 (40.3)
Better patient-reported health	429 (41.4)
No noticeable change	182 (17.6)
Worsening of symptoms	89 (8.6)
Other/unclear	5 (0.5)
Consultation with the patient's physician during deprescribing	
Always	402 (38.8)
Often	339 (32.7)
Sometimes	249 (24.0)
Never	46 (4.4)
Aware of AI- or software-based decision support tools for deprescribing	
Yes	701 (67.7)
No	335 (32.3)
Currently uses a digital tool to identify deprescribing opportunities	
Yes	641 (61.9)
No	395 (38.1)
Digital tools used to identify deprescribing opportunities	
Any AI chatbot	340 (32.8)
MedSafer	217 (20.9)
Beers Criteria app	131 (12.6)
STOPP/START-based tool	94 (9.1)
Other named digital resources/tools	39 (3.8)
None/no specific tool reported	82 (7.9)
Comfort using software/apps to assist medication review for deprescribing	
Very comfortable	399 (38.5)
Somewhat comfortable	516 (49.8)
Not at all comfortable	121 (11.7)
Perceived commonness of AI/computerized decision support for deprescribing	
Common	320 (30.9)
Occasional	367 (35.4)
Rare	257 (24.8)
Unknown	92 (8.9)

Data are presented as *n* (%) and percentages are based on *N* = 1,036. For multiple-response items ((pharmacist-reported perceived outcomes after deprescribing, digital tools used, and medication classes frequently deprescribed), respondents could endorse more than one option; therefore percentages may exceed 100%. The perceived-outcome items reflect pharmacists’ recollections and were not obtained directly from patients, linked to individual deprescribing events, or independently verified. Raw combined responses were decomposed into their main options and recounted. For the conditional item on tools used, blank follow-up responses were not shown; responses containing both “None” and a named tool were counted under the named tool(s) only, and “None/no specific tool reported” reflects standalone “None” responses. CPD, continuing professional development; AI, artificial intelligence; PPIs, proton-pump inhibitors; NSAIDs, non-steroidal anti-inflammatory drugs; HRT, hormone replacement therapy. For analytic classification, MedSafer was treated as a rule-based electronic clinical decision support system, Beers Criteria applications and STOPP/START-based tools were treated as criteria-based electronic resources, and only reported AI-chatbot use counted as AI use.

### Factors associated with AI use in deprescribing

Overall, 340 (32.8%) pharmacists were classified as AI users in deprescribing practice. Compared with non-users, AI users were more likely to work in urban settings (153, 45.0% vs. 262, 37.6%; *p* = 0.03), to report the availability of a private counselling area (261, 76.8% vs. 480, 69.0%; *p* = 0.01), and to report reliable internet access during working hours (268, 78.2% vs. 455, 65.4%; *p* < 0.001) (Table 2). No significant differences were observed according to pharmacy type, patient volume, prescription volume, years working in community pharmacy, or current professional position.

### Multivariable analysis of AI use in deprescribing

Male pharmacists had higher odds of AI use in deprescribing than female pharmacists (adjusted odds ratio [AOR] 1.32, 95% confidence interval [CI] 1.01–1.73; *p* = 0.042) (Table 4). Compared with pharmacists holding a BPharm/BSc degree, PharmD holders were more likely to report AI use (AOR 1.38, 95% CI 1.02–1.87; *p* = 0.038). Practicing in an urban location was independently associated with AI use when compared with rural practice (AOR 1.52, 95% CI 1.04–2.22; *p* = 0.031). Compared to pharmacists with no computer/tablet available, those reporting reliable internet access had substantially higher odds of AI use (AOR 3.89, 95% CI 1.95–7.76; *p* < 0.001).

**Table 4:** Multivariable logistic regression of using AI-chatbot in deprescribing-related practice across community pharmacists in Jordan.

Independent variables	AOR	95% Confidence interval		*P*-value
		Lower	Upper	
Gender				
Female	Reference			
Male	1.32	1.01	1.73	**0**.**042**
Region of pharmacy location				
Northern governorates	Reference			
Amman	1.21	0.87	1.68	0.254
Central governorates	1.08	0.68	1.72	0.738
Southern governorates	0.79	0.37	1.69	0.545
Highest pharmacy-related qualification				
BPharm/BSc Pharmacy	Reference			
PharmD	1.38	1.02	1.87	**0**.**038**
MSc in Pharmacy/Clinical Pharmacy	1.45	0.95	2.21	0.086
PhD in Pharmacy/Pharmaceutical Sciences	1.62	0.91	2.89	0.102
Other health-related degree	0.91	0.57	1.46	0.698
Area where the pharmacy is located				
Rural	Reference			
Semi-urban	1.24	0.85	1.81	0.267
Urban	1.52	1.04	2.22	**0**.**031**
Patients/customers served per working day				
< 50/day	Reference			
50–99/day	1.12	0.83	1.51	0.462
100–199/day	1.18	0.76	1.83	0.461
>=200/day	1.25	0.68	2.30	0.473
Prescription medicines dispensed per working day				
< 25/day	Reference			
25–49/day	1.08	0.78	1.49	0.645
50–99/day	1.24	0.82	1.88	0.308
100–199/day	1.31	0.72	2.38	0.376
>=200/day	1.42	0.71	2.84	0.322
Private counselling area available				
No	Reference			
Yes	1.29	0.96	1.73	0.094
Computer/tablet with internet access				
No computer/tablet available	Reference			
Computer/tablet without internet	1.58	0.71	3.52	0.264
Internet often unstable or slow	2.67	0.65	5.52	0.215
Reliable internet at all times	3.89	1.95	7.76	**<0**.**001**
Electronic system for prescriptions/records				
No, all on paper	Reference			
Yes, for some prescriptions	1.48	0.89	2.09	0.219
Yes, for all prescriptions	1.72	0.39	2.45	0.411
Use of online clinical resources				
Never	Reference			
Rarely	1.89	0.92	3.88	0.083
A few times per week	3.21	0.93	6.44	0.109
Several times per day	4.56	0.58	9.20	0.618

Variables with *P* < 0.30 in bivariate analysis were included in the multivariable model. Bold *P*-values indicate statistical significance (*P* < 0.05). Reference categories are indicated.

AOR, adjusted odds ratio; CI, confidence interval.

## Discussion

This cross-sectional study is among the first to oneself-reported use of AI in deprescribing-related practice among community pharmacists in Jordan. It shows that deprescribing-related activity is already common in Jordanian community pharmacy practice. More than three-quarters of pharmacists reported practicing deprescribing, nearly two-thirds reported usingat least one digital tool to identify deprescribing opportunities, and about one-third reported using AI specifically for deprescribing. These findings are important because earlier Jordanian literature generally described community pharmacies as highly accessible and well regarded by the public, but still largely centered on dispensing, with medication review and medication-reconciliation functions not yet fully embedded in routine care. In that context, the present results suggest that community pharmacists in Jordan are moving toward a more active medication-optimization role. However, because the digital-tool categories were non-mutually exclusive and the survey did not assess comparative preference, the difference between any digital-tool use and AI-chatbot use should be interpreted descriptively rather than as evidence that pharmacists favor conventional tools over AI (23, 24).

This study's high self-reported deprescribing prevalence appears higher than what previous regional data would have predicted. While previous Jordanian research found low awareness of medication reconciliation and only modest medication-history taking in community pharmacies (23), El-Dahiyat et al. (25), reported predominantly moderate deprescribing knowledge and practice in the UAE, with only approximately 44% of pharmacists reporting that they had intentionally practiced deprescribing. The estimate in the current research is likely greater for several reasons. First, rather than explicit awareness of potentially inappropriate drug criteria or a limited hospital-style reconciliation service, the present outcome captured any deprescribing-related behaviour in routine community practice. Second, during standard dispensing contacts, community pharmacists have the option to discontinue, taper, or refuse the prescription classes that were most frequently targeted in this research. Third, the lack of notable variations by workload, experience, or type of pharmacy implies that these opportunities are not limited to highly specialised settings but rather are prevalent in Jordanian community practice. This view also aligns with the Jordanian training literature, which continues to demonstrate restricted access to organised internal training; in other words, a substantial proportion of current deprescribing activity may be practice-driven and experiential rather than standardised within official services (14, 24).

Antibiotics were the most commonly targeted medication class, which suggests that some reported activity may reflect community-pharmacy antimicrobial stewardship rather than over deprescribing of chronic medicine. This result should not be taken as proof that formal deprescribing of long-term antibiotic treatment was widespread. Respondents may have used the term “deprescribing” to refer to antimicrobial-stewardship actions in community pharmacy encounters, such as challenging an unnecessary prescription, suggesting discontinuation or a shorter duration, refusing inappropriate non-prescription supply, or referring the patient or prescriber for review. Therefore, compared to the patient-centered withdrawal of long-term treatment described in the Introduction, the antibiotic discovery may represent a more expansive category of drug optimization. The high overall incidence of self-reported deprescribing may be partially explained by this wider interpretation, which also restricts direct comparison with studies that concentrate on older persons, polypharmacy, or possibly unsuitable chronic medications. Jordanian data suggest that community pharmacies are an important point of intervention in antibiotic supply and use (26). According to several Jordanian studies, PPIs are routinely recommended in general practice, and they are subjected to inappropriate and protracted usage, especially in the elderly (27, 28). International intervention studies found PPIs suitable for pharmacist-led deprescribing since tapering is straightforward and patients understand the risk-benefit balance of inappropriate usage (29, 30). The Canadian Deprescribing Network and other international gastroenterology groups report that PPIs are occasionally given without reason or for too long, causing polypharmacy and side effects (31). Our findings show a high rate of physician consultation during deprescribing, combined with pharmacists likely selecting lower-risk drug candidates This may explain the favourable self-reported outcomes observed, which is consistent with international evidence that pharmacist-led and decision-support-assisted deprescribing can reduce medications without short-term harm, though these outcomes were not independently verified (29, 32). However, the outcomes in the present study were based solely on pharmacists’ retrospective perceptions and were not independently verified; they should therefore be interpreted cautiously and not as evidence of causal clinical benefit.

Approximately two-thirds of the pharmacists in this study reported using at least one digital tool to identify deprescribing opportiunties, one-third specifically reported using AI chatbots, and one-fifth reported using MedSafer. Because respondents could select more than one tool and the questionnaire did not directly compare preference, usability, trust, or comfort across specific tools, these frequencies do not demonstrate that conventional digital resources were preferred over AI-assisted resources. This gap suggests that pharmacists are more comfortable with conventional digital resources than with AI-assisted decision making. It also suggests that low-threshold tools, such as chatbots or general online resources, may be easier to adopt than dedicated deprescribing platforms that require more structured data and workflow integration. Our interpretation partiallyaligns with prior Jordanian studies, which indicated that community pharmacists showed generally positive attitudes toward AI, but also reported important barriers including software and hardware limitations, technical difficulties, lack of competencies, resistance to change, cost, privacy concerns, and the need for human supervision (12, 19). It also fits earlier Jordanian ChatGPT data, in which many pharmacists had never used ChatGPT in practice despite perceiving potential benefit (13). In the present study, 32.8% of participants were categorized as utilizing an AI chatbot expressly to find deprescribing chances, whereas 67.2% were not. AI usage for deprescribing-related drug review was thus quantifiable, although it was not yet the predominant practice. The extended data collecting period, the inclusion of any AI chatbot rather than only ChatGPT, and the more focused emphasis on deprescribing might be the reasons for differences from the previous ChatGPT research; however, these arguments were not evaluated. Our findings are consistent with recent international evidence, where approximately one-third of US pharmacists reported using ChatGPT (33).

In a larger national survey of 1,363 practicing US pharmacists, 82.5% claimed some experience with AI, but only 38.7% had utilized it; big language models were the most often reported use, employed by 33.7% of respondents. Although 64.1% agreed that AI may boost professional performance and productivity, skepticism and anxiety about worker consequences remained popular (34). That study also indicated increased reported AI usage among male than female pharmacists, which is directionally similar with the link shown in our adjusted analysis, although the current finding should be taken carefully. High familiarity but uneven training was discovered in a different nationwide US study of 235 pharmacy faculty members and 405 pharmacy students; ethical issues and training were noted as the main obstacles to AI incorporation (35). In Eastern Romania, a recent cross-sectional study found favorable perceptions of AI-based applications for reducing medication errors, supporting counselling, and improving pharmacist–patient communication across community pharmacists (36).

A recent qualitative evidence from the UK highlights that AI tools for medication reviews must integrate seamlessly with existing systems and present actionable insights without overwhelming users, to support adoption among healthcare professionals (37).

In this study, the factors independently associated with AI usage support the hypothesis that preparedness drives adoption over routine workload. A reliable internet connection was the strongest correlate, which is expected because AI chatbots and web-based decision support are difficult to employ with poor connectivity. Urban pharmacies possess superior digital infrastructure, denser professional networks, and more clinical problem-solving opportunities, which may explain why they utilize AI more frequently. PharmD holders utilized AI to a greater extent than BPharm/BSc holders, potentially due to clinical experience and confidence in drug review decision-support systems. This aligns with international calls for integrating AI education into pharmacy curricula and professional development programs (38). The American Society of Health-System Pharmacists has emphasized that pharmacists with knowledge and experience in informatics are well-suited for designing, implementing, and researching AI applications (39). Even though this did not remain statistically significant after adjustment, pharmacies with the space and workflow to support confidential consultations may be more prepared to integrate discussion-based, AI-enabled medication reviews. AI utilization was not associated with years of practice, patient volume, prescription volume, or professional position, suggesting that Jordanian community pharmacy practice remains focused on connectivity, training, and the local practice environment. The minor association with the male sex may reflect unmeasured variations in technological exposure or self-efficacy rather than a persistent sex-based difference in practice. This finding should be interpreted cautiously given Jordan's female-dominated pharmacy workforce and may reflect sociocultural factors influencing technology engagement rather than inherent capability differences (40, 41).

This study has several implications for Jordanian practice and policy. First, community pharmacists appear ready to contribute meaningfully to deprescribing, but the service should be more clearly defined to distinguish stewardship-related antibiotic and over-the-counter (OTC) medicine decisions from formal deprescribing of chronic potentially inappropriate medications. Second, our findings suggest potential priorities fo community-pharmacy medication optimization in Jordan, including antimicrobial stewardship, chronic PPI review, rational supplement usage, safer analgesic use, and diabetes medication-intensity review. Third, because infrastructure and competence appear to influence AI uptake more than seniority or workload, implementation efforts should focus on reliable internet, interoperable digital records, practical deprescribing algorithms, AI governance, and continuing professional development (CPD) that combines clinical deprescribing skills with digital literacy. Finally, AI techniques should complement professional judgement and physician collaboration, particularly in community pharmacy, which is readily accessible yet structurally varied across locations.

### Limitations

The findings of this study should be interpreted carefully due to several limitations. First, Using overlapping internet, phone, and in-person channels, non-probability convenience sampling was used to recruit participants. The sample was not probability-based and should not be regarded as nationally representative, even though recruiting took place throughout many governorates. It is possible that younger, more tech-savvy, professionally connected, or clinically oriented pharmacists were more likely to take part. Additionally, independent pharmacies and pharmacists from northern governorates were overrepresented. Furthermore, because the recruiting channels overlapped and it was uncertain how many pharmacists got invitations overall, an overall response rate could not be determined. These characteristics restrict sectoral and geographic generalizability and may have led to overestimates of the adoption of digital tools and AI chatbots. Second, all metrics were self-reported, increasing recall and social-desirability bias. Pharmacists may have overstated their deprescribing, interprofessional collaboration, and AI tool utilization. Third, the phrase “deprescribing” could have been understood widely by respondents. Antibiotic-related responses might have been antimicrobial-stewardship actions rather than formal withdrawal of chronic therapy. Examples of such actions include questioning an unnecessary prescription, recommending discontinuation or a shorter duration, declining inappropriate non-prescription supply, or referring for prescriber review. Supplements and OTC analgesics may also be ambiguous. As a result, formal deprescribing as it is traditionally described may be overestimated in the stated prevalence and medication-class frequencies. Future research should identify antimicrobial stewardship and over-the-counter drug optimization from chronic medication deprescribing and provide clear, case-based criteria. Fourth, Fourth, Jordan is predominantly Arabic-speaking, whereas the questionnaire was administered only in English. Although pharmacy education is commonly delivered in English and the instrument underwent expert review and pilot testing, the absence of a formally translated and cross-culturally validated Arabic version may have affected comprehension for some eligible pharmacists and may limit measurement equivalence and generalizability. Fifth, the cross-sectional design precludes causal inference. It cannot establish whether digital readiness leads to AI usage or if pharmacists already predisposed to utilize AI are more likely to invest in digital infrastructure, nor how AI use affects deprescribing behaviours and patient outcomes over time. These concerns necessitate longitudinal investigation. Sixth, there were few responders who used certain digital tools, making it impossible to make reliable tool-specific comparisons. Rather of being an AI chatbot, MedSafer was categorized as a rule-based electronic clinical decision support system for the purposes of this research. Seventh, the stated post-deprescribing outcomes were not monitored using validated patient-reported outcome instruments, connected to specific deprescribing events, evaluated at predetermined follow-up intervals, or confirmed by patients, prescribers, medical records, or dispensing records. Baseline clinical state, the kind of deprescribing intervention, the extent or duration of change, concurrent therapy modifications, and other reasons were not recorded in the survey. Therefore, these results reflect the opinions of pharmacists and do not indicate that deprescribing led to better health, greater adherence, fewer side effects, or worsening of symptoms. Therefore, rather than being proof of therapeutic efficacy, outcome-related estimates should be taken as descriptive signals for further study. To evaluate the safety and efficacy of pharmacist-initiated or AI-supported deprescribing, prospective trials using patient-level data, predetermined end measures, adjudication, and suitable comparator groups are required. Finally, AI technology evolves rapidly; thus, the tools and adoption trends identified may shift over short periods, reducing the temporal generalizability of our results.

## Conclusion

The pharmacists who took part in this study reported that deprescribing-related activities were frequent in Jordan's community setting, with antibiotics, proton-pump inhibitors, and vitamins/supplements being the most popular targets. Approximately one-third reported using AI chatbots to help them make decisions about deprescribing. Pharmacists also reported perceived improvements in patient-reported health, adherence, and side effects. However, these outcomes were self-reported, not independently verified, and cannot be causally attributed to deprescribing or AI use. Reliable internet access was strongly associated with reported AI use, while male gender, PharmD qualification, and urban practice location showed smaller independent associations. These findings support the need for equitable digital infrastructure, continuing professional development, clear AI governance, and prospective controlled studies using objective patient-level measures.

## Data Availability

Data are available upon request. Requests to access the datasets should be directed to amaslamanie1095@gmail.com.
